# Circulating microRNAs as a Fingerprint for Endometrial Endometrioid Adenocarcinoma

**DOI:** 10.1371/journal.pone.0110767

**Published:** 2014-10-20

**Authors:** Lin Wang, Yan-Jie Chen, Kai Xu, Hua Xu, Xi-Zhong Shen, Rui-Qin Tu

**Affiliations:** 1 Department of Obstetrics and Gynecology, Shanghai Zhongshan Hospital, Fudan University, Shanghai, China; 2 Department of Gastroenterology, Zhongshan Hospital, Fudan University, Shanghai, China; University of Kentucky College of Medicine, United States of America

## Abstract

**Background:**

Endometrial cancer is the most common malignancy of the female genital tract worldwide, and endometrial endometrioid adenocarcinoma (EEC) is the major histological type of endometrial cancer. There is a great need for better markers with high sensitivity and specificity to permit early diagnosis and proper management of EEC. The aim of our study is to identify a miRNA classifier within plasma as a noninvasive biomarker for EEC diagnosis.

**Methods:**

This study was a retrospective case-control analysis which contained two independent cohorts including 93 participants. First, we screened 375 miRNAs in 29 plasma samples. 9 of the miRNAs were selected to be evaluated their expression by quantitative reverse-transcriptase polymerase chain reaction. A stepwise logistic regression model was then used to establish a new classifier in the validation cohort. Area under the receiver operating characteristic curve was used to evaluate the diagnostic accuracy. Co-expression analysis was used to verify the independence of results.

**Results:**

miR-15b, -27a, and -223 were found to be differentially expressed in the EEC plasma between the two cohorts and had few connections with other miRNAs. The areas under the curve (AUC) were 0.768, 0.813, and 0.768 for miR-15b, -27a, and 223, respectively. miR-27a and CA125 can be combined as a potential non-invasive biomarker for detecting EEC, with the AUC of 0.894.

**Conclusion:**

Our study demonstrated three miRNAs, including miR-15b, -27a, and -233 have a good clinical value in EEC diagnosis. The classifier, including miR-27a and CA125, demonstrated a high accuracy in the diagnosis of EEC and might serve as a novel non-invasive biomarker in the future.

## Introduction

Endometrial cancer is the most common malignancy of the female genital tract worldwide, and endometrial endometrioid adenocarcinoma (EEC) is the major histological type of endometrial cancer [1]. Its incidence has increased steadily due to changings in lifestyle of women, including dietary habits, delayed marriage and decreased gravidity, which make EEC become one of the major lethal of all gynecologic malignancies [2], [3]. Although the morphological alterations provide significant insights into EEC, it still has some limitations including morbidity and mortality. Currently the noninvasive diagnosis of EEC mainly relies on the combination of ultrasound, MRI, and serological markers, but none of them are completely satisfactory. Thus, the identification of accurate and validated prognostic biomarkers for EEC is needed to improve diagnosis, guide molecular targeted therapy, and lead better outcome of EEC.

Just one decade ago, a novel class of evolutionarily conserved small (18–24 nucleotides) non-coding RNA, or microRNAs (miRNAs) was discovered as important regulators of gene expression [4], [5]. Through partial homology to the 3′UTR in target mRNAs, miRNAs affect control of gene expression via repression of translation as well as reducing mRNA levels directly [4], [6]–[8]. Numerous studies have shown that alterations in miRNA expression may correlate with various pathological or physiological states. While a majority of miRNAs are detected intracellularly, lots of stable miRNAs have recently been found in circulation, and alterations of these extracellular miRNAs are tightly correlated with various diseases [9]–[11], suggesting the potential of miRNA signatures in disease diagnosis.

Our study performed genome-wide miRNA expression profiles (375 miRNAs) from plasma samples of EEC patients and normal controls, and the validation was conducted in an independent cohort including EEC patients, patients with other endometrial diseases (including endometrial polyps, atypical hyperplastic endometrium), and normal controls. We demonstrated miR-15b,-27a, and -233 has a great clinical value in diagnosing EEC, and a panel, which was combined with miR-27a and CA125, has a considerable clinical value in diagnosing EEC.

## Materials and Methods

### Study design and participants

Our study was approved by the ethics committee (ethics committee of Zhongshan Hospital of Fudan University, Shanghai), and written informed consent was obtained from all study participants. The present study was divided into two phases: (1) Discovery phase. In this phase, global miRNAs profiling were screened in 29 samples using quantitative real-time PCR reaction. A Mann-Whitney test was used to discover differentially expressed miRNAs. (2) Validation phase. Nine candidates discovered were further validated in plasma samples of 64 participants by quantitiative RT-PCR (qRT-PCR). Three of them, which were found to be differentially expressed, were evaluate the diagnostic performance by area under the ROC (receiver operating characteristic) curve (AUC). (3) All the data of the participants in the validation phase were used to construct the diagnostic model by logistic regression.

As shown in Table 1, a total of 93 people participated in this study, including 40 EEC patients, 19 patients with endometrial polyps, 4 atypical hyperplastic endometrium patients, and 30 healthy individuals. The inclusion and exclusion criteria of the patient cohorts include: (a) having a distinctive pathologic diagnosis of EEC, (b) having no anticancer treatment before the blood sample collection, (c) having no other serious illness such as heart attacks, renal inadequacy, leukemia, hepatitis, and so on, (d) having no injury of peritoneal or abdominal organ. No patient underwent the relapse or death of the cancer a half year after the surgery.

**Table 1:** Characteristics of the whole participants.

		Discovery phase		Validation phase	
		EEC	Control	EEC	Control
**n**		9	20	31	33
**Age (years)**		67.0 (55, 72)	50.9 (21, 69)	62.4 (48, 83)	59.6 (31, 82)
**CA125 (U/ml)**	**<35**	7	20	20	33
	**>35**	2	0	11	0
**TNM stage**	**I–II**	8	–	22	–
	**III–IV**	1	–	9	–
**Differentiation**	**G1–G2**	7	–	22	–
	**G3**	2	–	9	–
**Pelvic effusion**	**+**	1	0	8	0
	−	8	20	24	33
**Body mass index (Kg/m^2^)**	**<27**	3	7	10	13
	**>27**	6	13	21	20

### Sample processing and miRNA isolation

For plasma preparation, the whole blood (2 ml) was collected into EDTA tubes. Within 1 h at 4°C, the tubes were subjected to centrifuge at 820 g for 10 min at 4°C. The resulting plasma was transferred into new clear tubes, followed by a 10-min high-speed centrifugation at 16,000 g at 4°C to pellet the remaining cellular debris. Supernatant was transferred to clear tubes and stored at −80°C.

We extracted miRNA from 500 µL plasma by using miRcute miRNA Isolation Kit according to the instruction from the manufacturer (Tiangen, Beijing, China). After the lysate was mixed into the plasma, cel-miR-39 (5*10^−3^ pmol) was added into the samples as the spike-in controls. The concentration was quantified by NanoDrop 1000 Spectrophotometer (NanoDrop Technologies, Waltham, MA). The concentration of miRNA extrated from plasma ranged from 10 to 20 ng/µL.

### Quantitative real-time PCR (qPCR) of miRNAs

One hundred and forty nanogram plasma miRNA was polyadenylated by poly (A) polymerase in a 20-µL volume, and 6 µL of the poly (A) reaction solution was reverse transcribed to cDNA in another 20-µL volume using the Sharpvue miRNA First Strand Kit (Biovue, Shanghai, China) to generate the first-strand cDNA. The reaction solution including cDNA products was diluted to one tenth with RNase free water and stored at −20°C before downstream quantitative PCR analysis. The single tube miRNA assays were used to detect and quantify mature miRNAs by Sharpvue 2× Universal qPCR Master Mix High Rox kit (Biovue, Shanghai, China) and Sharpvue Human miRNA Primer Array kit (Biovue, Shanghai, China) per the instructions in the kits. Each sample was detected by 375 miRNA primers and one spike-in miRNA (cel-miR-39) primers. miRNAs expression levels were quantified using ABI 7900HT Fast Real-Time PCR System (Applied Biosystems, USA). The reactions were incubated in a 384-well optical plate at 95°C for 2 min, followed by 3 cycles of 96°C for 5 s and 60°C for 1 min, 37 cycles of 96°C for 5 s and 60°C for 30 s, and running melting curve at the end. SYBR green dye and Rox dye were used as reporter and reference, respectively. The relative expression of each miRNA was calculated from the equation 2^−ΔCT^ with cel-miR-39 as the spike-in control to normalize the data. △CT was calculated by subtracting the CT values of cel-miR-39 from the CT values of the miRNAs of interest.

### Statistical analysis

Data were analyzed using SPSS (version 19.0). For the data obtained by qRT-PCR, the nonparametric Mann-Whitney test was used for the comparison between EEC and control. A stepwise logistic regression was used to establish a model as a surrogate marker to diagnose the EEC. AUC was used as an accuracy index for evaluating the diagnostic performance of the significant miRNAs.

### Processing the miRNA expression data

The miRNAs were divided into two groups, and the Pearson correlation coefficients of the miRNA expression were calculated between each pairs. The co-expression miRNA pair was defined as correlation coefficient larger than 0.75 and P<0.05 [12]. The top 500 co-expression miRNA pairs were exhibited by CytoScape [13].

## Results

### Global plasma miRNA profiling and data analysis

We adapted a quantitative real-time PCR reaction for 375 human miRNAs to screen significant differential expression levels of miRNAs between EEC and control groups. A Mann-Whitney test was used to discover differentially expressed miRNAs. Four miRNAs, including miR-223, -143, -15b, and -27a, were significantly upregulated in EEC group (fold change = 5.5–6.5; P<10^−7^), while another five miRNAs, including miR-1179, -4638-5p, -4665-5p, -3145-5p, and -4502 were significantly downregulated in EEC group (fold change = 0.04–0.2; P<1.3×10^−4^, Table 2). In summary, nine differentially expressed miRNAs were identified as candidates for further investigation.

**Table 2:** Candidate miRNAs from discovery phase result.

	ECC vs Control		
	P value	Fold change	regulation
hsa-miR-1179	4.39393E-07	0.041255	down
hsa-miR-4638-3p	1.57508E-09	0.111331	down
hsa-miR-4665-5p	1.88429E-05	0.123958	down
hsa-miR-3145-5p	0.000127123	0.150915	down
hsa-miR-4502	2.16271E-05	0.17775	down
hsa-miR-27a	3.45679E-08	5.633507068	up
hsa-miR-15b	7.16484E-08	6.096250655	up
hsa-miR-143	7.22628E-08	6.209981148	up
hsa-miR-223	1.73472E-09	6.409200686	up

### Differential expression profile of nine selected microRNAs

**Figure 1:**
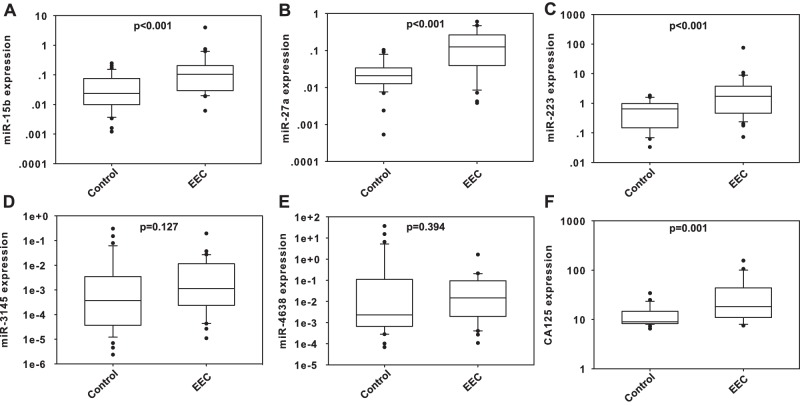
Plasma levels of miR-15b, -27a, -223, -3145, -4638 and CA125 in validation phase. Significant up-regulation of miR-15b, -27a, -223, and CA125 (A, B, C, and F) can be found in EEC patients, while there’s no statistically significant differences in the level of miR-3145 and -4638 (D and E) between the EEC and control. EEC: endometrial endometrioid adenocarcinoma.

We then tested the expression of nine candidated miRNAs which were selected from the previous step in an independent cohort of 64 plasma samples. miR-143, -179, -4665-5p, and -4502 had a low level in the plasma and cannot be detected in all of the samples. Five miRNAs, including miR-223, -15b, -27a, -3145-5p, and -4638-5p, had a high level in the plasma (Fig. 1). Among these five miRNAs, miR-223, -15b, and -27a passed the quality control and showed a significantly different expression levels between the EEC and control groups, including endometrial polyps, atypical hyperplastic endometrium, and normal controls. We also showed the differential expression of CA125 (Fig. 1F).

### ROC curve analysis of selected miRNAs and CA125

**Figure 2:**
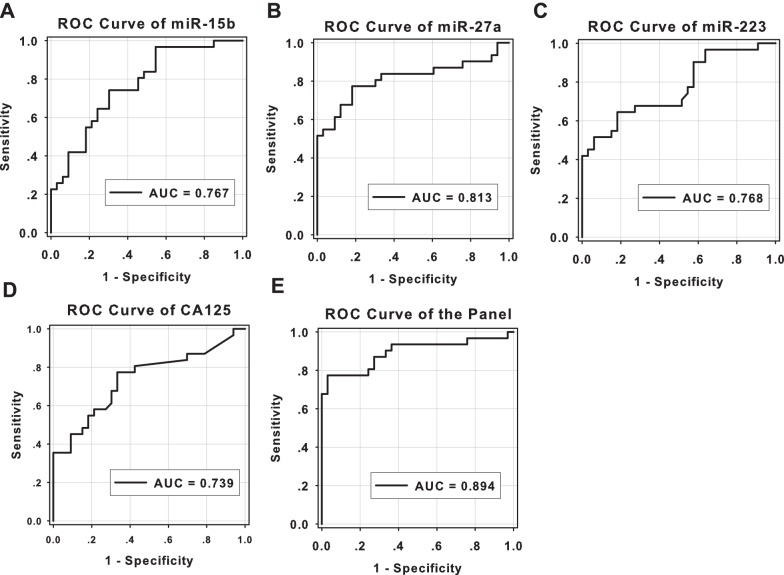
ROC curve analysis for miR-15b (A), -27a (B), -223 (C), CA125 (D), and the panel (E). ROC: receiver operating characteristic; AUC: area under the ROC curve.

To further evaluate the diagnostic value of the three miRNAs, ROC curves were then constructed to estimate the sensitivity and specificity of these three miRNAs and CA125. The AUC were 0.768 (95% CI 0.653, 0.882; sensitivity = 0.742, specificity = 0.697), 0.813 (95% CI 0.699, 0.927; sensitivity = 0.774, specificity = 0.818), and 0.768 (95% CI 0.651, 0.885; sensitivity = 0.645, specificity = 0.818) for miR-15b, 27a, and 223, respectively, while only 0.739 (95% CI 0.614, 0.863; sensitivity = 0.774, specificity = 0.667) for CA125 (Fig. 2A, B, C, D).

### Establish the logit model as a surrogate marker

To further evaluate the diagnostic value of the combination of the miRNAs and CA125, a stepwise logistic regression analysis (with forward LR method) was applied on the validation data set. miR-27a and CA125 could be combined as a potential classifier for detecting EEC, and the formula of the classifier was as follow: 0.097×*CA125*+23.931×*miR-27a*-3.308. The ROC curve of the panel has an AUC of 0.894 (95% CI, 0.807, 0.980; sensitivity = 0.774, specificity = 0.970) (Fig. 2E).

### Exhibit the co-expression miRNA network

**Figure 3:**
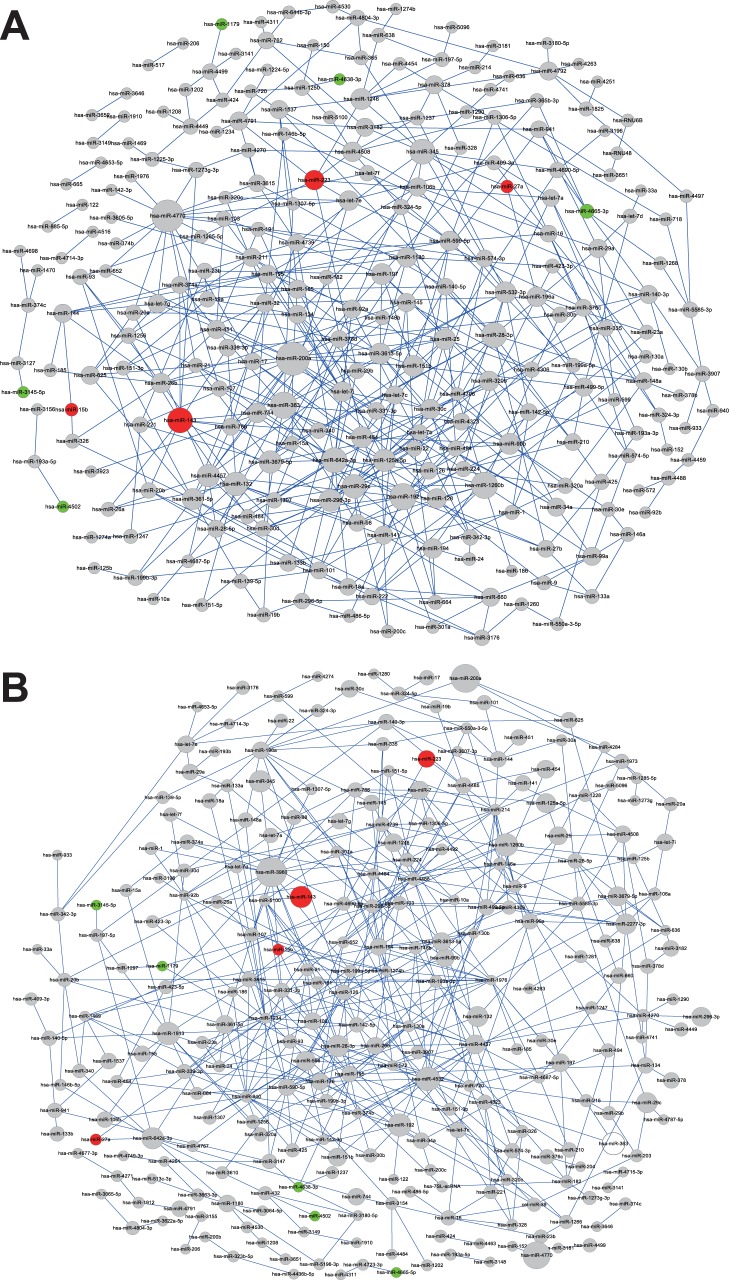
The co-expression miRNA network in plasma of EEC (A) and normal people (B). The miRNAs expressed differentially in the plasma are color-coded (red, upregulated; green, downregulated). The size of the nodes is related with correlation degree.

We generated a top500 co-expression miRNA network for cancer (Fig. 3A) and normal control (Fig. 3B). It is important to note that, to build this co-expression network, we used all the 375 miRNAs as input rather than only the nine differentially expressed miRNAs. In cancer plasma, the most connected hub was has-miR-200a (degree 17), followed by has-miR-4770 (degree 16), whereas in nonmalignant plasma, has-miR-3960 (degree 18) was the most connected node. It was interested to found that the connection degree was only 2, 3, and 2 in plasma of normal people, and 7, 2, and 2 in plasma of EEC patients for miR-223, 15b, and 27a respectively.

## Discussion

Endometrial cancer is the most common malignancy of the female genital tract worldwide, and EEC is the major histological type of endometrial cancer. Currently the noninvasive diagnosis of EEC mainly relies on the combination of ultrasound, MRI, and serological markers, but none of them are completely satisfactory. CA125 has been used for many years as a serum marker for endometrial cancer diagnosis and screening. However, it has been recognized that CA125 levels has poor specificity in the detection of endometrial cancer. Other gynecologic malignancies such as ovarian cancer can also show the raise of CA125. There is a great need for better markers with high sensitivity and specificity to permit early diagnosis and proper management of EEC.

A large number of miRNAs are proved to be present in circulation. They exhibit different profiles in patients with diverse diseases, including acute myocardial infarction [14], [15], ectopic pregnancy [16], rheumatic diseases [17], liver cirrhosis [11], and various types of malignancies [9], [18], [19]. All these findings bear a resemblance to circulating miRNAs serving as a reliable noninvasive biomarker for the detection of diseases. The present study demonstrated miR-15b, -27a, and -233 had a great clinical value in diagnosing EEC, and we also developed an unprecedented classifier which could be a set of potential non-invasive marker for diagnosing EEC. The classifier, combined miR-27a and CA125, demonstrated high accuracy in the diagnosis of EEC. The association at the tissue level between EEC and the three miRNAs has not been reported yet. But other studies showed that these three miRNAs might related with the neoplasia. Kim and his colleagues found that platelet-derived growth factor (PDGF) signaling pathway regulates smooth muscle cell (SMC)- specific gene expression and cell proliferation by modulating the expression of miR-15b to induce a dedifferentiated state in the vascular smooth muscle cells (VSMCs) [20]. Vimalraj and his colleagues found that miR-15b can act as a positive regulator for osteoblast differentiation [21]. It was found that miR-27a functioned as a tumor promoter in various cancers through targeting MAP2K4 [22], AGGF1 [23], prohibitin [24], and other genes that regulate specificity protein transcription factors and the G2-M checkpoint [25]. The miR-27a was also targeted by genistein [26], anticancer agent [27]. miR-223 was found promoted the growth and invasion of tumor cells by targeting tumor suppressor PAX6 [28] and FBXW7/hCdc4 [29], [30]. The co-expression analysis in our research exhibited that the connection degree was only 2, 3, and 2 in plasma of normal people, and 7, 2, and 2 in plasma of EEC patients for miR-223, 15b, and 27a respectively, which indicates that these three miRNAs might have their unique signal pathway to regulate the development of EEC. Further functional study is needed to confirm the role of these three miRNAs in EEC.

It has been found that the circulating miRNAs has the capability to diagnose EEC according to the previous studies. Jia W. and his colleagues found a four-miRNA signature, including miR-222, -23, -186, and -204), might serve as a non-invasive approach for EEC diagnosis [31]. It is the first and only study to identify a serum miRNA-based EEC signature using a genome-wide serum miRNA expression profiling analysis. In our research, we confirmed that miR-223 has a high value in serving as a diagnostic biomarker. But we also found that miR-15b and -27a had a high value in serving as a diagnostic biomarker. On the other hand, the combination of miR-27a and CA125 had an AUC of 0.894 (95% CI, 0.807, 0.980; sensitivity = 0.774, specificity = 0.970), which could be an optimal non-invasive biomarker to diagnose EEC. In the co-expression analysis, these three miRNAs had few connections with other miRNAs, indicated that they would hardly be influenced by the expression change of other miRNAs and could be stable biomarkers for EEC.

miRNAs in the circulation are extremely stable and protected from degradation by RNase activity or harsh environmental factors by microparticles [9]. Microparticles may transfer their components and contents to the selected target cells. Through this way, they can mediate cell activation, metabolism, phenotypic modification, and reprogramming of cell function [32], [33]. We found miR-15b, -27a, and -223 significantly upregulated in the plasma of EEC patients, which might leak from the cancer cells, or might be packed into microvesicles and actively secreted into the blood circulation and activated the anti-tumor mechanisms in the body. The effects of upregulated miRNAs in the circulation of EEC patients need to be further investigated.

Compared with other studies of circulating miRNAs in diagnosing EEC, our study is unique as the following reasons. First of all, we used not only healthy samples as controls, but also other endometrial diseases (including endometrial polyps, atypical hyperplastic endometrium) into the research to avoid the risk of indistinguishable of EEC from other endometrial diseases. Secondly, considering there’s no standard endogenous control for the circulating miRNA studies, we used the spike-in control (cel-miR-39) in the study, which leaded to a highly reliable conclusion. Last but not least, one particular miRNA in circulation may help to distinguish between patients and controls, but lots of the researches would choose a panel of miRNAs to raise the sensitivity and specificity, which make the experimental result hard to be applied in clinical circumstance. Our study found that the combination of miR-27a and CA125 demonstrated high accuracy in diagnosis of EEC with an AUC of 0.894. This result includes only one new index; therefore reduces the test cost effectively.

Although the miRNA classifier had a high diagnostic value in our research, there are still some limitations. First of all, as the difficulty in sample collection, the number of patients with atypical hyperplastic endometrium prevented us from testing in a more detailed classification. Studies including larger sample size are needed in the future. Secondly, considering the limited number of studies and the high cost of assay, it is still hard to extend the miRNA as a routine diagnostic biomarker into the clinical application. More researches and efforts are needed to change the experimental findings into clinical applications.

In summary, our study demonstrated three miRNAs, including miR-15b, -27a, and -233 has a great clinical value in diagnosing EEC. The classifier, including miR-27a and CA125, demonstrated high accuracy in the diagnosis of EEC and might serve as a novel non-invasive biomarker in the future.

## References

[1] Zhang Y, Wang J (2010) Controversies in the management of endometrial carcinoma. Obstetrics and gynecology international 2010: 862908. 20613958 10.1155/2010/862908 PMC2896852

[2] Amant F, Moerman P, Neven P, Timmerman D, Van Limbergen E, et al (2005) Endometrial cancer. Lancet 366: 491–505. 16084259 10.1016/S0140-6736(05)67063-8

[3] Di Cristofano A, Ellenson LH (2007) Endometrial carcinoma. Annual review of pathology 2: 57–85. 10.1146/annurev.pathol.2.010506.091905 18039093

[4] Bartel DP (2004) MicroRNAs: genomics, biogenesis, mechanism, and function. Cell 116: 281–297. 14744438 10.1016/s0092-8674(04)00045-5

[5] Ambros V (2004) The functions of animal microRNAs. Nature 431: 350–355. 15372042 10.1038/nature02871

[6] Yekta S, Shih IH, Bartel DP (2004) MicroRNA-directed cleavage of HOXB8 mRNA. Science 304: 594–596. 15105502 10.1126/science.1097434

[7] Ambros V (2001) microRNAs: tiny regulators with great potential. Cell 107: 823–826. 11779458 10.1016/s0092-8674(01)00616-x

[8] He L, Hannon GJ (2004) MicroRNAs: small RNAs with a big role in gene regulation. Nature reviews Genetics 5: 522–531. 10.1038/nrg1379 15211354

[9] Mitchell PS, Parkin RK, Kroh EM, Fritz BR, Wyman SK, et al (2008) Circulating microRNAs as stable blood-based markers for cancer detection. Proceedings of the National Academy of Sciences of the United States of America 105: 10513–10518. 18663219 10.1073/pnas.0804549105 PMC2492472

[10] Ichikawa D, Komatsu S, Konishi H, Otsuji E (2012) Circulating microRNA in digestive tract cancers. Gastroenterology 142: 1074–1078 e1071. 10.1053/j.gastro.2012.03.008 22433392

[11] Chen YJ, Zhu JM, Wu H, Fan J, Zhou J, et al (2013) Circulating microRNAs as a Fingerprint for Liver Cirrhosis. PloS one 8: e66577. 23805240 10.1371/journal.pone.0066577 PMC3689750

[12] Liu X, Liu ZP, Zhao XM, Chen L (2012) Identifying disease genes and module biomarkers by differential interactions. Journal of the American Medical Informatics Association: JAMIA 19: 241–248. 22190040 10.1136/amiajnl-2011-000658 PMC3277635

[13] Kohl M, Wiese S, Warscheid B (2011) Cytoscape: software for visualization and analysis of biological networks. Methods Mol Biol 696: 291–303. 21063955 10.1007/978-1-60761-987-1_18

[14] D'Alessandra Y, Devanna P, Limana F, Straino S, Di Carlo A, et al (2010) Circulating microRNAs are new and sensitive biomarkers of myocardial infarction. European heart journal 31: 2765–2773. 20534597 10.1093/eurheartj/ehq167 PMC2980809

[15] Devaux Y, Vausort M, Goretti E, Nazarov PV, Azuaje F, et al (2012) Use of circulating microRNAs to diagnose acute myocardial infarction. Clinical chemistry 58: 559–567. 22252325 10.1373/clinchem.2011.173823

[16] Zhao Z, Zhao Q, Warrick J, Lockwood CM, Woodworth A, et al (2012) Circulating microRNA miR-323-3p as a biomarker of ectopic pregnancy. Clinical chemistry 58: 896–905. 22395025 10.1373/clinchem.2011.179283 PMC3694411

[17] Alevizos I, Illei GG (2010) MicroRNAs as biomarkers in rheumatic diseases. Nature Reviews Rheumatology 6: 391–398. 20517293 10.1038/nrrheum.2010.81 PMC3041596

[18] Yu SL, Chen HY, Chang GC, Chen CY, Chen HW, et al (2008) MicroRNA signature predicts survival and relapse in lung cancer. Cancer cell 13: 48–57. 18167339 10.1016/j.ccr.2007.12.008

[19] Baker SG (2009) Improving the biomarker pipeline to develop and evaluate cancer screening tests. Journal of the National Cancer Institute 101: 1116–1119. 19574417 10.1093/jnci/djp186 PMC2728744

[20] Kim S, Kang H (2013) miR-15b induced by platelet-derived growth factor signaling is required for vascular smooth muscle cell proliferation. BMB reports. 10.5483/BMBRep.2013.46.11.057 PMC4133843 24152911

[21] Vimalraj S, Partridge NC, Selvamurugan N (2014) A Positive Role of microRNA-15b on Regulation of Osteoblast Differentiation. Journal of cellular physiology. 10.1002/jcp.24557 PMC4038448 24435757

[22] Pan W, Wang H, Jianwei R, Ye Z (2014) MicroRNA-27a Promotes Proliferation, Migration and Invasion by Targeting MAP2K4 in Human Osteosarcoma Cells. Cellular physiology and biochemistry: international journal of experimental cellular physiology, biochemistry, and pharmacology 33: 402–412. 10.1159/000356679 24556602

[23] Xu Y, Zhou M, Wang J, Zhao Y, Li S, et al (2014) Role of microRNA-27a in down-regulation of angiogenic factor AGGF1 under hypoxia associated with high-grade bladder urothelial carcinoma. Biochimica et biophysica acta 1842: 712–725. 24462738 10.1016/j.bbadis.2014.01.007

[24] Liu T, Tang H, Lang Y, Liu M, Li X (2009) MicroRNA-27a functions as an oncogene in gastric adenocarcinoma by targeting prohibitin. Cancer letters 273: 233–242. 18789835 10.1016/j.canlet.2008.08.003

[25] Mertens-Talcott SU, Chintharlapalli S, Li X, Safe S (2007) The oncogenic microRNA-27a targets genes that regulate specificity protein transcription factors and the G2-M checkpoint in MDA-MB-231 breast cancer cells. Cancer research 67: 11001–11011. 18006846 10.1158/0008-5472.CAN-07-2416

[26] Xu L, Xiang J, Shen J, Zou X, Zhai S, et al (2013) Oncogenic MicroRNA-27a is a target for genistein in ovarian cancer cells. Anti-cancer agents in medicinal chemistry 13: 1126–1132. 23438830 10.2174/18715206113139990006

[27] Chintharlapalli S, Papineni S, Abdelrahim M, Abudayyeh A, Jutooru I, et al (2009) Oncogenic microRNA-27a is a target for anticancer agent methyl 2-cyano-3,11-dioxo-18beta-olean-1,12-dien-30-oate in colon cancer cells. International journal of cancer Journal international du cancer 125: 1965–1974. 19582879 10.1002/ijc.24530 PMC2766353

[28] Huang BS, Luo QZ, Han Y, Li XB, Cao LJ, et al (2013) microRNA-223 promotes the growth and invasion of glioblastoma cells by targeting tumor suppressor PAX6. Oncology reports 30: 2263–2269. 23970099 10.3892/or.2013.2683

[29] Li J, Guo Y, Liang X, Sun M, Wang G, et al (2012) MicroRNA-223 functions as an oncogene in human gastric cancer by targeting FBXW7/hCdc4. Journal of cancer research and clinical oncology 138: 763–774. 22270966 10.1007/s00432-012-1154-x PMC11824240

[30] Kurashige J, Watanabe M, Iwatsuki M, Kinoshita K, Saito S, et al (2012) Overexpression of microRNA-223 regulates the ubiquitin ligase FBXW7 in oesophageal squamous cell carcinoma. British journal of cancer 106: 182–188. 22108521 10.1038/bjc.2011.509 PMC3251856

[31] Jia W, Wu Y, Zhang Q, Gao G, Zhang C, et al (2013) Identification of four serum microRNAs from a genome-wide serum microRNA expression profile as potential non-invasive biomarkers for endometrioid endometrial cancer. Oncology letters 6: 261–267. 23946815 10.3892/ol.2013.1338 PMC3742699

[32] Mause SF, Weber C (2010) Microparticles: protagonists of a novel communication network for intercellular information exchange. Circulation research 107: 1047–1057. 21030722 10.1161/CIRCRESAHA.110.226456

[33] Lee TH, D'Asti E, Magnus N, Al-Nedawi K, Meehan B, et al (2011) Microvesicles as mediators of intercellular communication in cancer–the emerging science of cellular ‘debris'. Seminars in immunopathology 33: 455–467. 21318413 10.1007/s00281-011-0250-3

